# An interactive surgical planning tool for acetabular fractures: initial results

**DOI:** 10.1186/1749-799X-5-50

**Published:** 2010-08-04

**Authors:** Jürgen Fornaro, Marius Keel, Matthias Harders, Borut Marincek, Gábor Székely, Thomas Frauenfelder

**Affiliations:** 1 Institute of Diagnostic Radiology, University Hospital of Zurich, Zurich, Switzerland; 2 Department of Orthopaedic surgery, University of Berne, Inselspital, Berne, Switzerland; 3 Computer Vision Lab, ETH Zurich, Switzerland

## Abstract

**Background:**

Acetabular fractures still are among the most challenging fractures to treat because of complex anatomy, involved surgical access to fracture sites and the relatively low incidence of these lesions. Proper evaluation and surgical planning is necessary to achieve anatomic reduction of the articular surface and stable fixation of the pelvic ring. The goal of this study was to test the feasibility of preoperative surgical planning in acetabular fractures using a new prototype planning tool based on an interactive virtual reality-style environment.

**Methods:**

7 patients (5 male and 2 female; median age 53 y (25 to 92 y)) with an acetabular fracture were prospectively included. Exclusion criterions were simple wall fractures, cases with anticipated surgical dislocation of the femoral head for joint debridement and accurate fracture reduction. According to the Letournel classification 4 cases had two column fractures, 2 cases had anterior column fractures and 1 case had a T-shaped fracture including a posterior wall fracture.

The workflow included following steps: (1) Formation of a patient-specific bone model from preoperative computed tomography scans, (2) interactive virtual fracture reduction with visuo-haptic feedback, (3) virtual fracture fixation using common osteosynthesis implants and (4) measurement of implant position relative to landmarks. The surgeon manually contoured osteosynthesis plates preoperatively according to the virtually defined deformation. Screenshots including all measurements for the OR were available.

The tool was validated comparing the preoperative planning and postoperative results by 3D-superimposition.

**Results:**

Preoperative planning was feasible in all cases. In 6 of 7 cases superimposition of preoperative planning and postoperative follow-up CT showed a good to excellent correlation. In one case part of the procedure had to be changed due to impossibility of fracture reduction from an ilioinguinal approach. In 3 cases with osteopenic bone patient-specific prebent fixation plates were helpful in guiding fracture reduction. Additionally, anatomical landmark based measurements were helpful for intraoperative navigation.

**Conclusion:**

The presented prototype planning tool for pelvic surgery was successfully integrated in a clinical workflow to improve patient-specific preoperative planning, giving visual and haptic information about the injury and allowing a patient-specific adaptation of osteosynthesis implants to the virtually reduced pelvis.

## Introduction

Acetabular fractures still are among the most challenging fractures to treat because of complex anatomy, involved surgical access to fracture sites and the relatively low incidence of these lesions [1], resulting in long learning curves. Primary goals of acetabular surgery are anatomic reduction of the articular surface with attention to careful soft tissue management, facilitating rapid postoperative recovery with early rehabilitation and a long-term functioning hip joint [2]. Proper evaluation and surgical planning is necessary to achieve these goals [1].

The ilioinguinal and the posterior Kocher-Langenbeck approaches with or without surgical hip dislocation are the most commonly used operative approaches for the treatment of pelvic and acetabular fractures [3-5]. In 1994 Cole introduced the modified Stoppa approach as an alternative for the ilioinguinal approach, allowing access to essentially the entire pelvic ring through a single window [6-8]. Some centres have developed less invasive modifications of these approaches or implemented percutaneous screw fixation techniques following open or closed reduction for distinct fracture patterns [9-12], reducing damage from soft tissue dissection. Especially when using such minimally invasive techniques a careful planning of the operative approach as well as type, size and placement of osteosynthesis implants is crucial and may decrease the operative time. Patients may also benefit from decreased blood loss, decreased fluoroscopy radiation exposure, more accurate plate and screw placement and lowered incidence of neurovascular complications.

Today high scanner speeds and diagnostic accuracy superior to other modalities has made computed tomography (CT) imaging the standard for evaluation of blunt trauma to the pelvis [13,14]. Multiplanar reformatted images and volume rendered views [15] of the CT datasets are readily available on current workstations. These 2D and 3D visualizations are complementary in fracture classification, identifying the main fracture fragments and recognizing their displacement and rotation as well as their spatial relation. But because of their static nature they may give only limited insight into the optimal choice of surgical approach and osteosynthesis implants for internal fixation [16,17]. Thus surgeons still have to make some important decisions based on the mental combination of available imaging studies, or sometimes intraoperatively after fracture fragment reduction, using intraoperative fluoroscopy as a flexible, yet limited 2D imaging modality.

During the last years a few preoperative planning tools specific to acetabular fractures have been developed, leveraging advances in radiology and computer technology. Cimerman et al. reported favorable results in the preoperative planning of pelvic and acetabular fracture reduction and osteosynthesis using a commercially available tool with a mouse-based interface comparable to Computer-Aided Design (CAD) software [18]. The surgeons performed the virtual operations themselves after patient-specific virtual models had been built from CT datasets by computer engineers. Brown et al. fabricated life-size wax stereolithographic replica of the fractured hemipelvis and the reversed non-fractured hemipelvis to prebend fixation plates and to produce methyl methacrylate drill guidance templates matching the planned screw trajectories [16]. They could achieve accurate plate and screw placement using this technique.

The goal of this study was to test the feasibility of preoperative surgical planning in acetabular fractures using a new prototype planning tool based on an interactive virtual reality-style environment, including fracture reduction, fixation and measurement.

## Materials and methods

### Patients and Data Acquisition

From June 2007 to March 2008 7 patients (5 male and 2 female) with a median age of 53 y (range: 25 to 92 y) were prospectively included (Table 1). Inclusion criterion was diagnosis of an acetabular fracture, excluding simple wall fractures as well as cases with anticipated surgical dislocation of the femoral head for joint debridement and accurate fracture reduction. Informed consent was obtained from all patients.

**Table 1:** Patient data

Case	Age (yr)/Gender	Acetabular fracture type
1	25/F	both column (left)
2	48/M	anterior column (left)
3	56/M	both column (left)
4	33/M	anterior column (right)
5	53/F	T-shaped (left)
6	82/M	both column (left)
7	92/M	both column (left)

All patients underwent a whole body CT scan (Sensation 64, Siemens Medical Solutions, Forchheim, Germany) on the day of admission according to standardized trauma protocol. Near-isotropic axial-oriented CT images with a slice thickness of 1 mm were reconstructed using a bone kernel for sharp depiction of bone fragment edges. Data were transferred to a picture archiving and communication system (PACS, Agfa HealthCare, Dübendorf, Switzerland).

### Model Generation

A commercially available software package (Amira 3.1, TGS Europe, Paris, France) was used for semiautomatic segmentation of the pelvic bones and fracture fragments. Pelvic bone and bone fragment surfaces were extracted using the Generalized Marching Cubes algorithm [19], generating triangulated surface models with 100'000 triangles for each patient. The procedure was performed by a radiologist (J.F.). In addition we built a library of models of differently sized trauma reconstruction plates and screws currently used at our hospital for acetabular fracture fixation. Models of osteosynthesis implants were based on tetrahedral volume meshes with approximately 10'000 tetrahedra for an average sized reconstruction plate.

### Surgical Planning Tool

**Figure 1:**
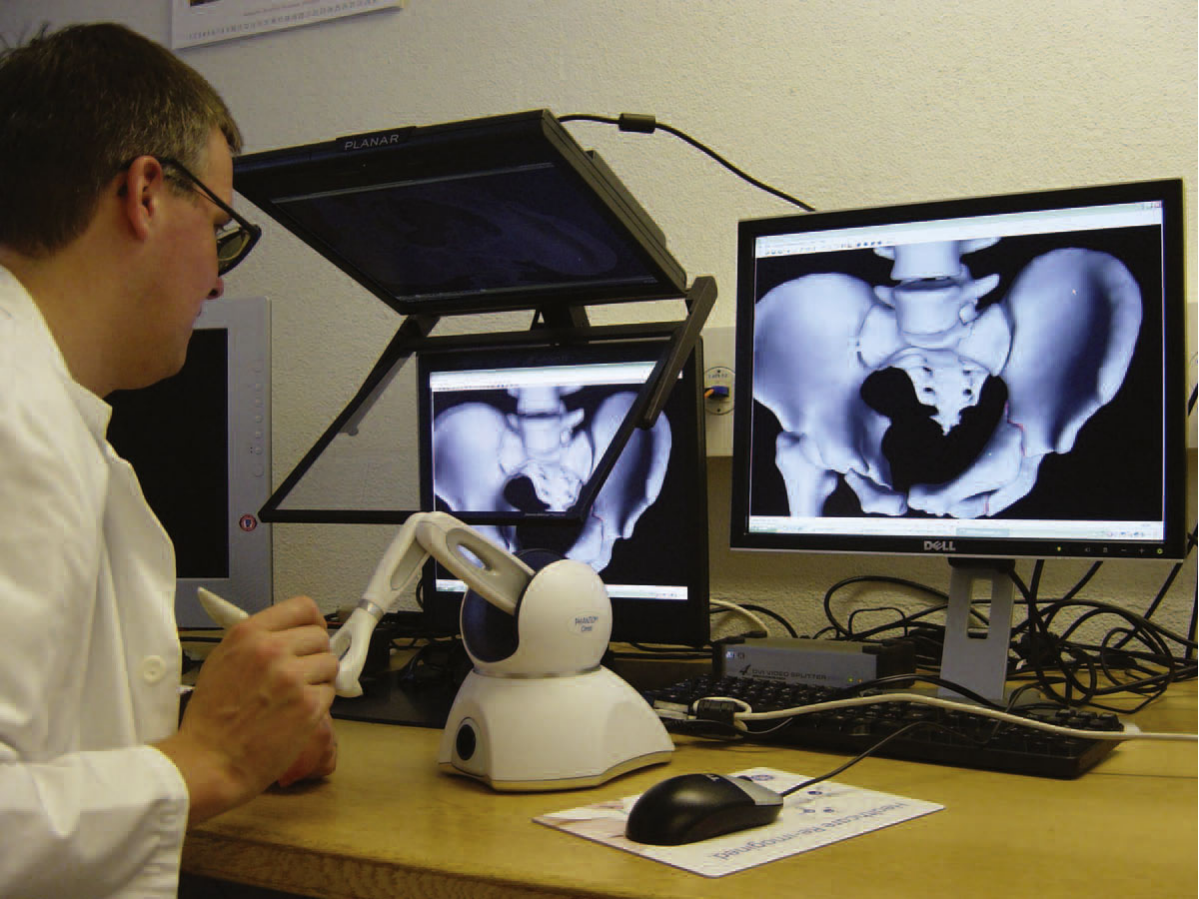
**Setup showing a haptic device and the 3D monitor**.

The generated models of pelvic bones and bone fragments were imported into our planning tool in the common STL or Wavefront OBJ file formats. The tool was developed in-house in the C++ programming language, using OpenGL for graphical and the PHANTOM Omni^® ^Developer Kit (SensAble Technologies, Woburn, MA, USA) for haptic rendering. It runs on a commercially available personal computer running Microsoft Windows. For haptic user interaction we chose the relatively low-cost PHANTOM Omni^® ^Haptic Device allowing for 6 degrees of freedom positional sensing (translation and rotation) as well as 3 degrees of freedom positional force feedback (only translation). In addition an implemented stereo rendering using a stereoscopic monitor (Planar Systems, Beaverton, OR, USA) was implemented (Figure 1) [20].

### Interactive Fracture Reduction

**Figure 2:**
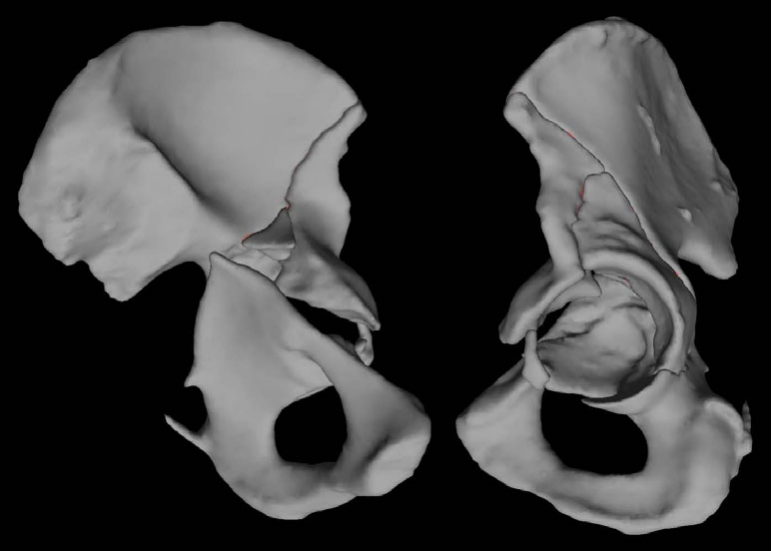
**Case 6 - model of the left hemipelvis on the day of admission shows a fracture of the left acetabulum involving both the anterior and posterior columns**. Oblique medial and lateral views.

In the planning tool, the user can interact with the virtual pelvic bones and bone fragments through the haptic device, by dragging and rotating them into anatomically correct positions (Figure 1 and 2). This process is supported by visual as well as haptic feedback in order to achieve precise bone fragment positioning. The overlaps of colliding bone fragments are visually encoded by locally changing surface colours. In addition, restoring forces resulting from bone fragment collisions are rendered to the haptic device.

Reference segmentation was performed by a radiologist (J.F.) in preparation for the fracture fixation planning done by the surgeon (M.K.). Nevertheless the surgeon was able to experiment with fracture fragment reduction to gather information about spatial relations of the fragments.

### Adaptation of osteosynthesis implants

**Figure 3:**
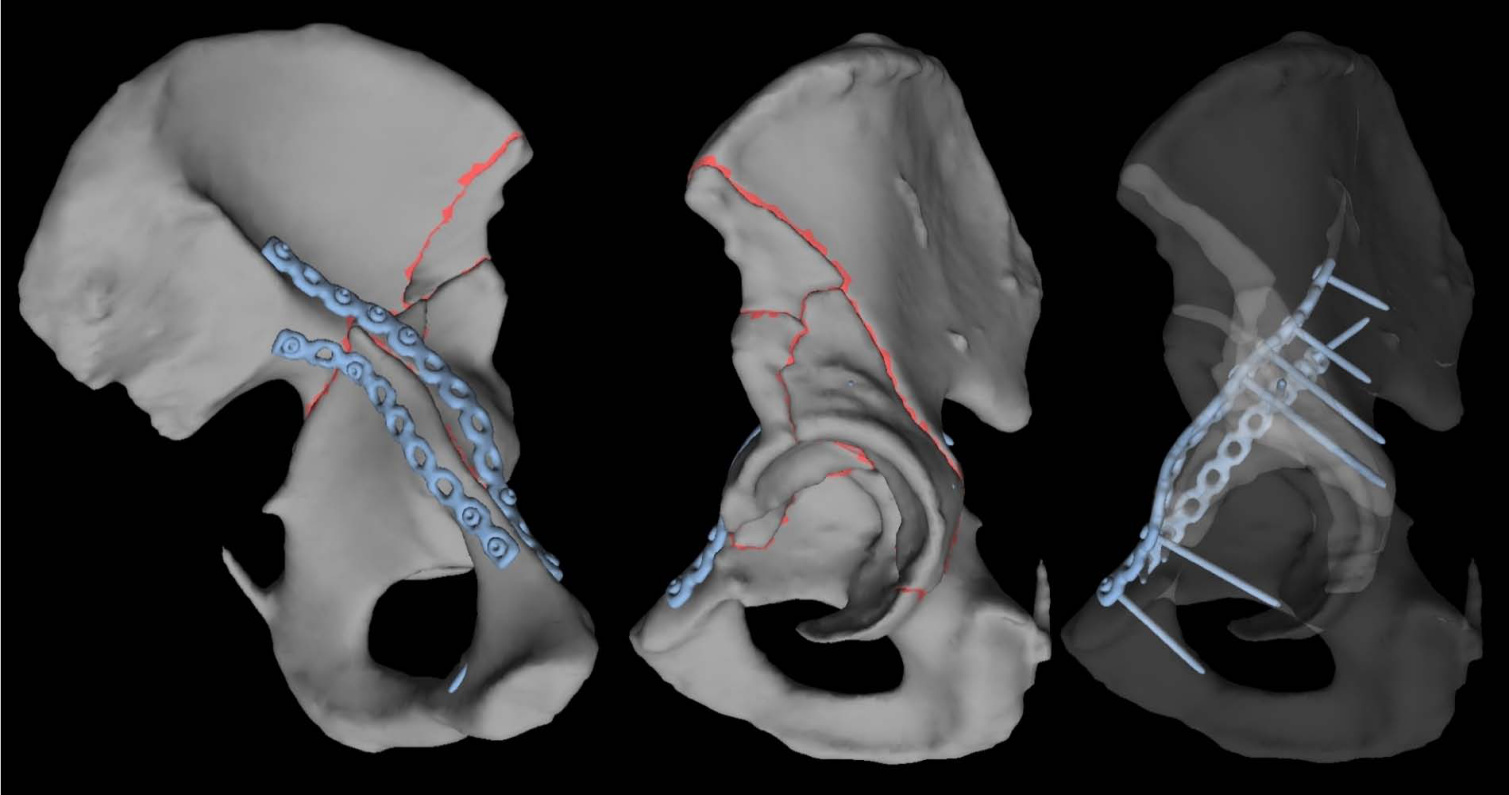
**Case 6 - model of the left hemipelvis after virtual fracture reduction and adaptation of osteosynthesis implants**. Oblique medial and lateral views.

In a second step the planning system allows the adaptation of appropriate osteosynthesis implants onto the reduced virtual pelvis. The user first draws a sketch of the desired plate placement directly onto the bone surface using the haptic device cursor. The system then automatically contours the tetrahedral model of a reconstruction plate of a user-selected type onto the virtual bone surface according to this sketch. Thereafter the user can place screws of different lengths either through plate holes at angles restricted by the type of implant or freely into the pelvic bone. Figure 3 shows a rendering of the model of the left hemipelvis after adaptation of osteosynthesis implants. The additional file 1 depicts the entire procedure.

### Preoperative contouring of osteosynthesis implants

Measurements like angles and lengths in 3D space were taken in relation to specific landmarks visible or palpable on the pelvic bone during the operation. Finally a report was generated including relevant screenshots, executed measurements, type and size of osteosynthesis implants as well as bending and torsion angles of fixation plate segments in all three planes. The surgeon (M.K.) used this information to manually contour osteosynthesis implants preoperatively according to this report. Additional screenshots were exported to the PACS and loaded on a screen in the OR as necessary.

### Evaluation

Time needed for building the patient-specific models from CT datasets, for virtual fracture reduction and fixation as well as the operative time was measured. In all patients a follow-up CT was performed 2 to 4 days after surgery. Congruence of the acetabular joint surface was determined according to Matta [5]: displacement of 1 mm or less was considered an anatomic reduction, of 2 to 3 mm a satisfactory and greater than 3 mm an unsatisfactory reduction.

Qualitative visual analysis of the accuracy of internal fixation was done by means of hybrid renderings of the postoperative CT and respective preoperative planning, after manually registering the pelvic bones into the same space. Placement of osteosynthesis implants was then compared on these renderings.

## Results

According to the Letournel classification [21] there were 4 cases with both column fractures, 2 cases with anterior column fractures and 1 case with a T-shaped fracture including a posterior wall fracture (Table 1).

### Segmentation and Mesh Generation

The segmentation of the fracture was the most time consuming part of the preoperative planning. Part of the segmentation had to be performed manually due to a large number of fracture fragments in 6 patients and osteopenic bone or severely impacted fragments in 3 patients. The median time needed to segment the osseous parts of an entire pelvis and to extract a surface model was 130 minutes (range 83 to 221 minutes).

### Interactive Fracture Reduction

Compared to a standard CAD-style mouse-based user interaction the haptic device integrated in our setup allowed a more direct and intuitive manipulation of fracture fragments. Median time of 8 minutes (range 6 to 15 minutes) was measured for fracture reduction by a trained user (J.F.).

### Preoperative planning and operative outcome

A Stoppa approach combined with the first window of the ilioinguinal approach was planned and executed in five cases and an ilioinguinal approach in one case. In one case a combined Stoppa and posterior approach was planned and executed (Table 2). The planned fracture fixation was followed completely in six cases and partially in one case (case 5).

**Table 2:** Procedure, used osteosynthesis implants and articular displacement comparing pre- to postoperative CT

Case	Surgical approach	Fixation	Articular dis-placement (mm)
1	Stoppa, first window of ilioinguinal approach	prebent 14-hole and 5-hole plates	3
2	Ilioinguinal approach	prebent 9-hole plate, 7.3 mm lag screw (acetabular dome)	1
3	Stoppa, first window of ilioinguinal approach	prebent 12-hole plate, two 7.3 mm lag screws (posterior column)	1
4	Stoppa, first window of ilioinguinal approach	prebent 12-hole plate, 7.3 mm lag screw (acetabular dome)	1
5	Stoppa, additional posterior approach	prebent 9-hole and 7-hole plates, additional 5-hole plate	3
6	Stoppa, first window of ilioinguinal approach	prebent 12-hole and 9-hole plates	2
7	Stoppa, first window of ilioinguinal approach	prebent 12-hole and 9-hole plates	2

**Figure 4:**
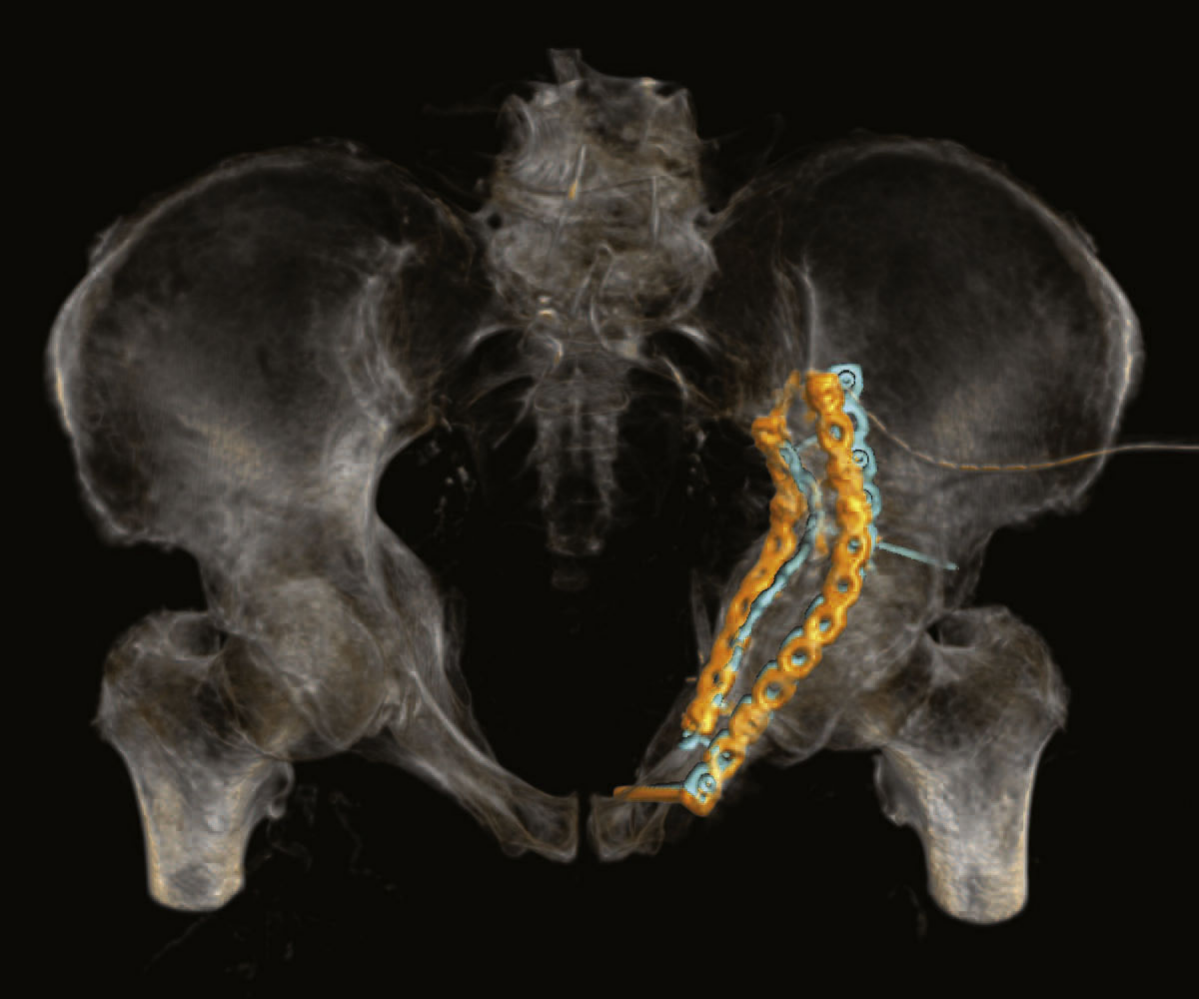
**Case 6 - hybrid rendering of the postoperative CT and preoperative planning showing the osteosynthesis implants as planned (blue) and as executed (orange)**. Inlet view.

**Figure 5:**
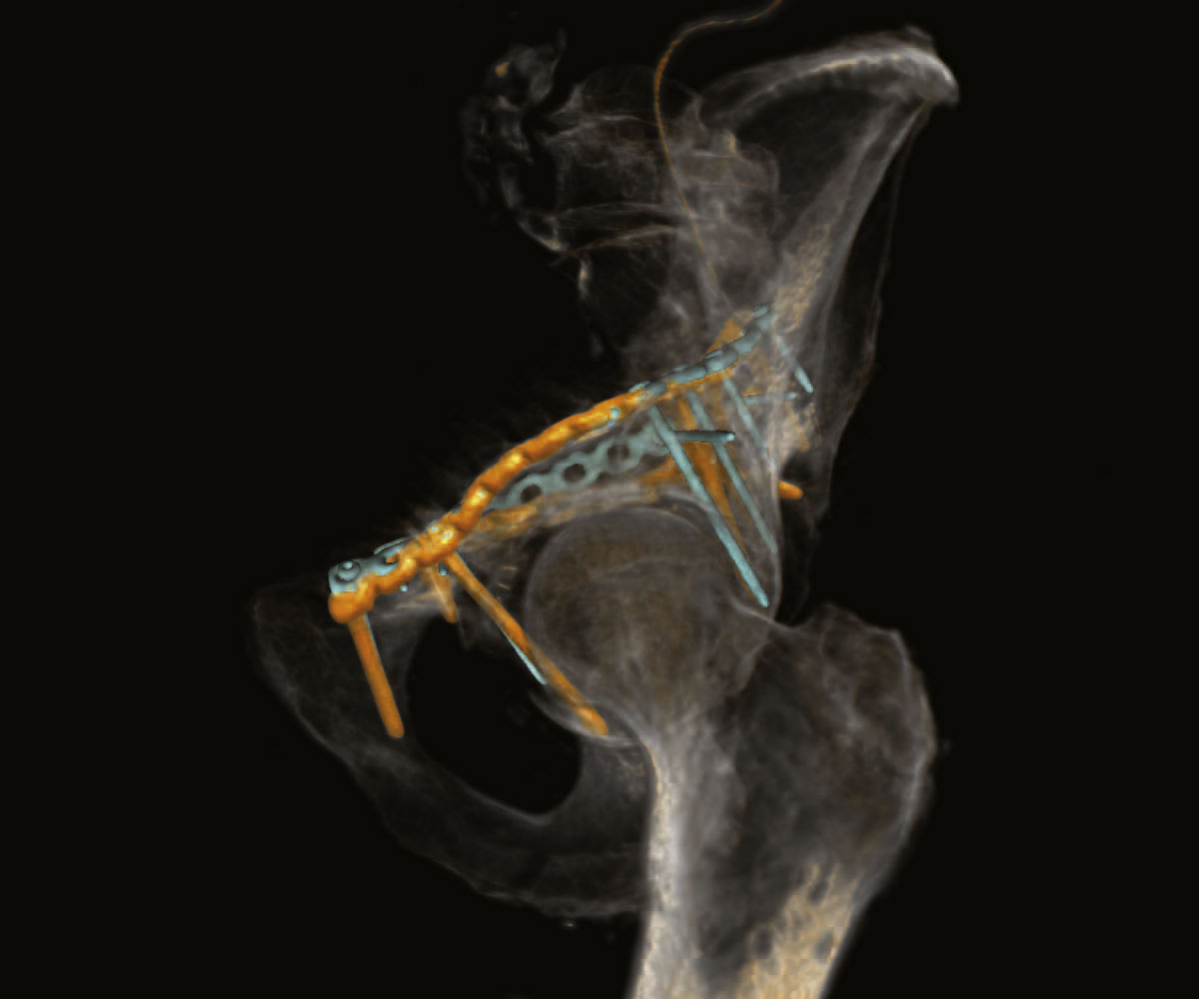
**Case 6 - hybrid rendering of the postoperative CT and preoperative planning showing the osteosynthesis implants as planned (blue) and as executed (orange)**. Oblique lateral view.

**Figure 6:**
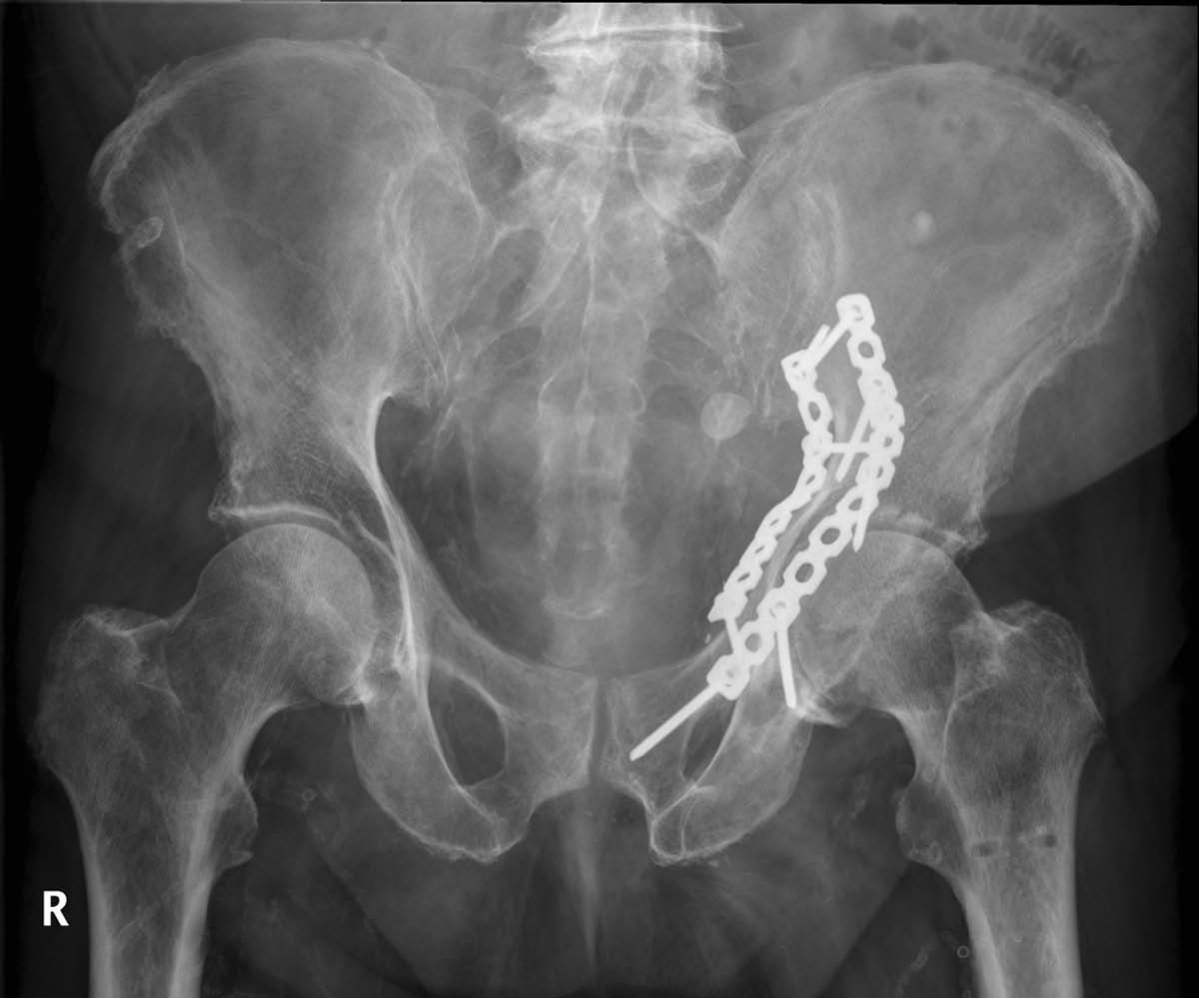
**Case 6 - antero-posterior radiograph of the postoperative result**.

In case 6 (Figure 4, 5 and 6), placement of the fixation plate on the acetabular dome shows a very good match between planning and actual execution while the second plate on the quadrilateral surface could not be placed exactly as planned. Because soft tissue was interfering with the placement of the screws, the plate had to be tilted slightly.

**Figure 7:**
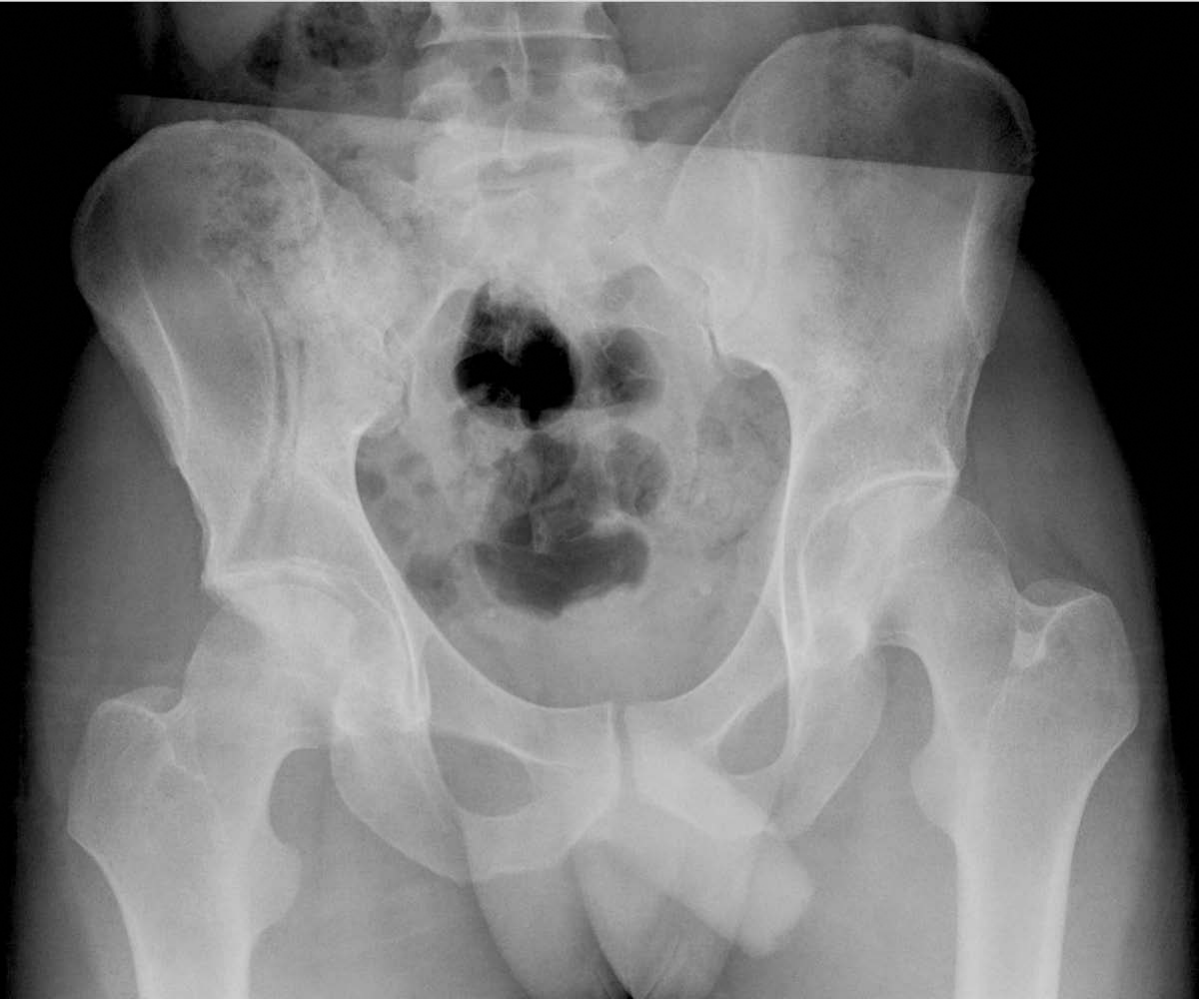
**Case 4 - antero-posterior radiograph on the day of admission shows an anterior column fracture of the right acetabulum**.

**Figure 8:**
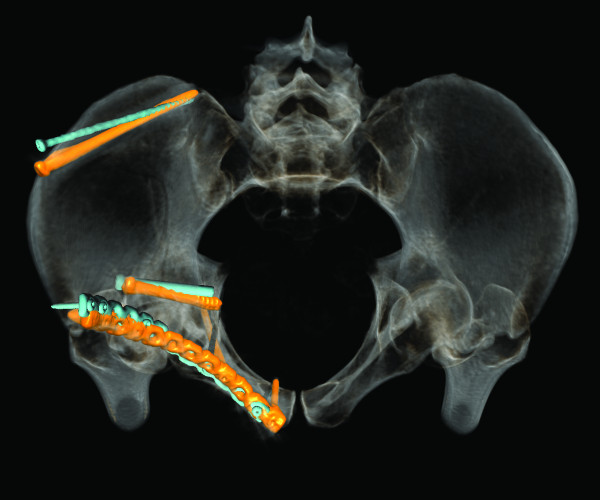
**Case 4 - hybrid rendering of the postoperative CT and preoperative planning showing the osteosynthesis implants as planned (blue) and as executed (orange)**. Inlet view.

**Figure 9:**
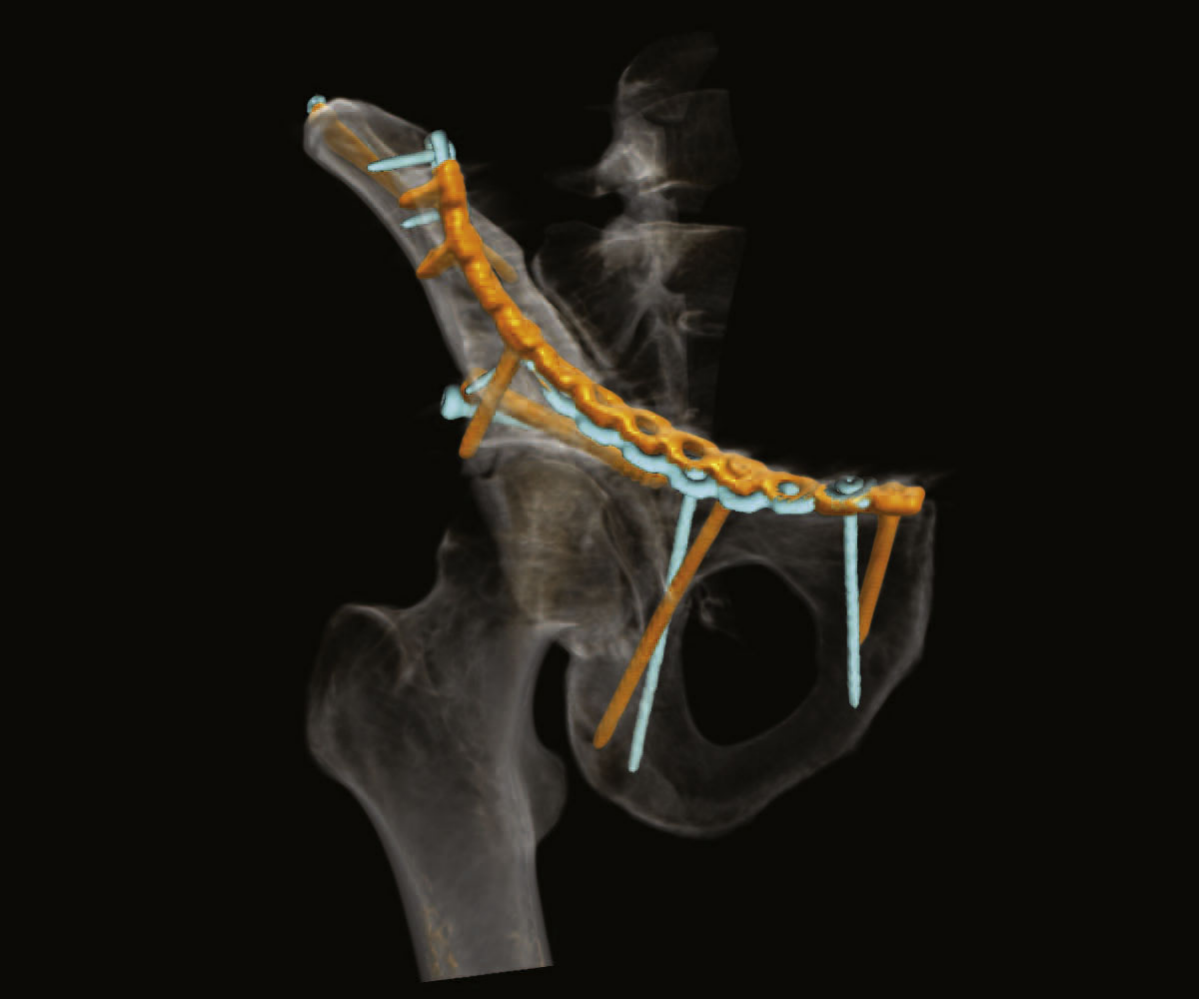
**Case 4 - hybrid rendering of the postoperative CT and preoperative planning showing the osteosynthesis implants as planned (blue) and as executed (orange)**. Oblique lateral view.

**Figure 10:**
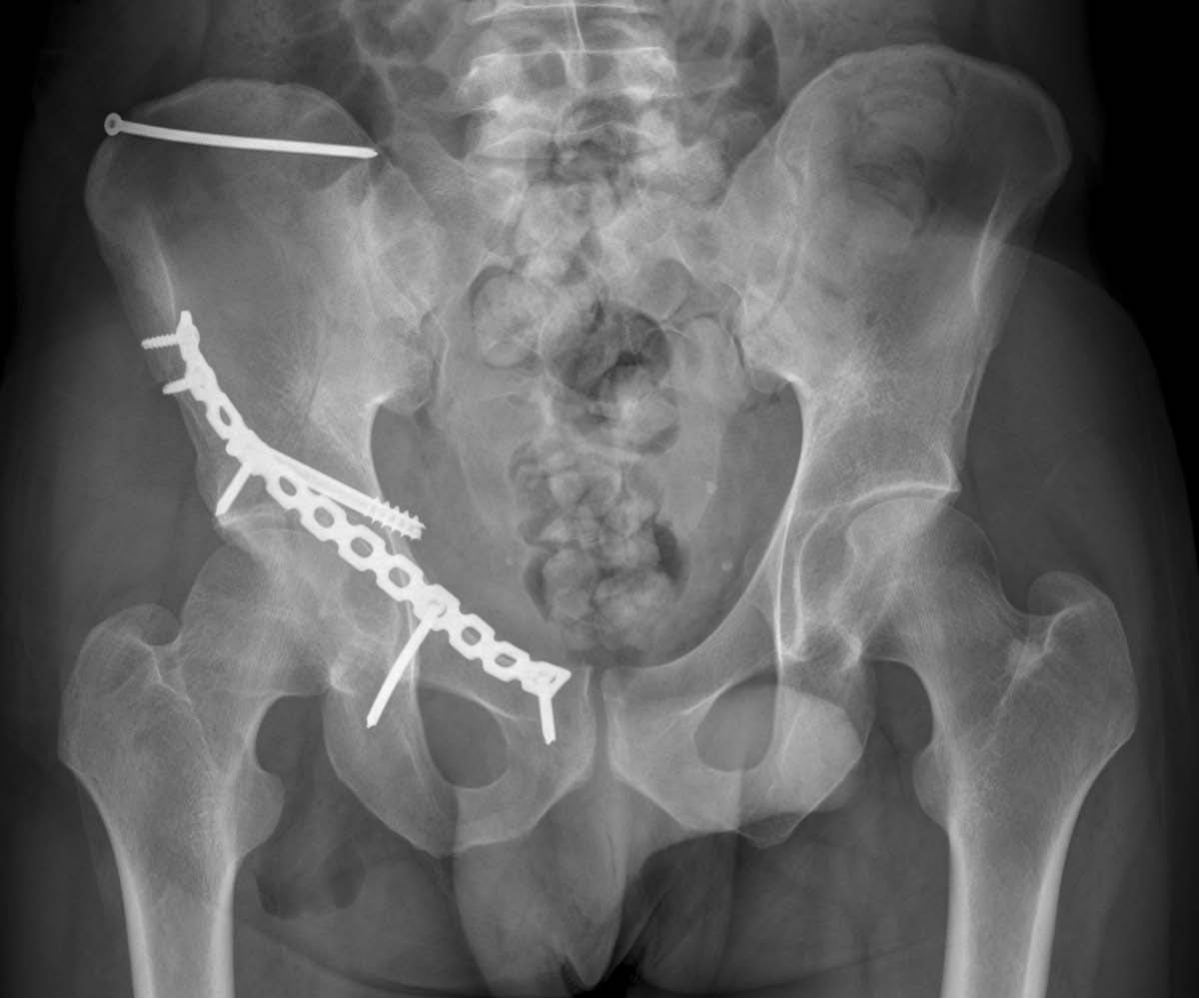
**Case 4 - antero-posterior radiograph of the postoperative result**.

Special attention was given to complement conventional internal fixation with percutaneous screw fixations. For example fixation of a posterior column fracture after reduction from an anterior approach was performed, avoiding an additional posterior approach. In one case percutaneous screw fixation of the posterior column (case 3) and in three cases of the dome of the acetabulum was successfully planned and performed (cases 2, 4 and 6). In case 4 complementary screw fixation of the acetabular dome after a both column fracture was performed (Figure 7, 8, 9 and 10).

Prebent fixation plates were used in all cases. In four cases (cases 2, 3, 6 and 7) with severely comminuted injuries to the pelvis this tremendously helped in guiding the fracture reduction.

Comparing the postoperative follow-up CT scans to respective preoperative planning, a good correlation was found in six of seven cases. The remaining case (case 5) partially failed due to the impossibility to reduce the fracture in the planned manner.

Postoperative congruence of the acetabular joint surface as determined according to Matta [5] in the follow-up CT was anatomic in three cases (43%) and satisfactory in four cases (57%) (Table 2). There was no case with inadvertent penetration of the hip joint.

We found no serious postoperative complications such as deep infections or failure of osteosynthesis implants. Analysis of functional outcome, for example occurrence of posttraumatic osteoarthritis of the hip joint, has not been included in this study because of the absence of long-term follow-up.

## Discussion

Acetabular fractures are severe injuries, often occurring in polytrauma patients as a result of a high-energy trauma such as motor vehicle accidents or falls from a height [22]. Less often they occur as a result of a minor trauma in older patients presenting with osteopenic bone [23].

Anatomic reduction of the acetabulum and stable fixation are primary goals in acetabular trauma surgery. Open reduction and internal fixation with several available approaches [3-5,7,8] remains the standard for definitive treatment, while in recent years less invasive modifications and minimally invasive percutaneous techniques have been developed [9-12].

Definitive treatment with open reduction and internal fixation typically is performed three to five days after the injury to prevent excessive bleeding that can be found in acute pelvic surgery [8]. This implies that there is enough time for meticulous preoperative surgical planning.

Cimerman et al. introduced a surgical planning software for pelvic and acetabular fractures with a mouse-based CAD-style interface [18]. In contrast, the presented tool was designed with a virtual reality-style visuo-haptic interface, generating an artificial sense of touch for the surgeon to more naturally interact with fracture fragments in a 3D environment and to simulate relevant steps of the operative procedure. Despite the rapid advances in radiology and computer technology in the last years and developments in minimally invasive surgery, surgical simulation and planning is rarely used in clinical routine. There are different reasons for the slow adoption of such technologies. One important factor may be the reservation of surgeons to explore new technologies as they are devoted to their technical skills and performance. Yet we think that with the maturing of a new generation of surgeons amenable for new technologies and with the introduction of tools implementing more intuitive interfaces, the integration of such technologies will accelerate.

The emphasis in designing the presented tool was not on execution of a surgical technique, but on supporting the preoperative surgical planning. The developed planning software consists of three consecutive steps: virtual fracture reduction and internal fixation using patient-specific CT data as well as measurement and documentation.

The tool enabled fast and reliable virtual fracture reduction. Interactive manipulation of the fracture fragments gave the surgeon insight into their spatial relation and helped in choosing the operative approach. Citak et al. showed that virtual planning of acetabular fracture reduction helps in understanding the fracture morphology and leads to more accurate and efficient reductions [17].

Virtual internal fixation allowed contouring models of osteosynthesis implants currently used at our hospital to the reduced pelvis. According to measured bending and torsion angles between plate segments the surgeon could bend the fixation plates preoperatively. The use of prebent fixation plates adjusted to the patient-specific anatomy and fracture pattern was found to be extremely helpful in guiding fracture reduction especially of severely comminuted acetabular injuries, pushing the fracture fragments into their anatomic position while tightening the screws.

Finally the tool also supported us in planning minimally invasive percutaneous screw fixations in selected fracture patterns. Screws should be placed as perpendicular as possible to the fracture plane while maintaining a safe distance to the hip joint. To enable the most accurate application of minimally invasive planning in the operating room, different measurements like angles and lengths in 3D space were taken in relation to specific landmarks visible or palpable on the pelvic bone.

In this study, the planned fracture fixation was followed completely in six cases and partially in one case with a good to satisfactory radiographic result according to Matta [5] in all cases. In the cases a good correlation between preoperative planning and respective postoperative follow-up CT scans was found. In particular no case with inadvertent penetration of the hip joint was observed. In one case the surgical planning partially failed due to the impossibility to execute fracture reduction as planned preoperatively. In a further case the fixation plate could not be placed on the quadrilateral surface exactly as planned, because of soft tissue interfering with the placement of the screws. The plate consequently had to be tilted slightly with screw trajectories directed more caudally as planned (Figure 4 and 5).

A first limitation of this study is the limited number of patients. Also due to the variability of injury patterns, it is difficult to make definite quantitative conclusions. This study therefore only is able to show initial experiences and a larger patient population is requested to further assess the presented tool.

A second limitation is the time-consuming segmentation of the pelvic bones and fracture fragments for the generation of the patient-specific models, requiring manual refinements especially in osteopenic bone or severely impacted fractures. In this study, segmentation was performed by a radiologist but could also be performed by a trained technician or surgeon. In addition, further developments in segmentation algorithms will accelerate or even automate this task.

As a final limitation, we did not simulate interfering soft tissues with the current design of the presented tool. Soft tissue structures like muscles and tendons inserting into pelvic bones, blood vessels and pelvic organs were not modelled. In reality these structures interfere with fracture reduction and narrow down the working space or can even render a desired fracture fixation impossible.

In conclusion, the presented prototype software tool for surgical planning of acetabular fractures gives visual and haptic information about the injury and allows a patient-specific adaptation of osteosynthesis implants to the virtually reduced pelvis. Manual prebending of fixation plates according to the procedure plan can guide fracture reduction especially in severely comminuted injuries.

In future the coupling of the presented planning tool with an intraoperative guiding system will be planned, enhancing the transfer of the surgical planning into the operating room.

In addition the information of the shape of the planned plate can be exported in STL-format enabling to order a prebent plate from dedicated companies.

## Competing interests

The authors declare that they have no competing interests.

## Authors' contributions

JF designed the study and programmed the software. MK carried out the read-out and recruited the patients. MH participated in programming the software. BM and GS edited the manuscript and participated in the study design. TF conceived the study, participated in its design, wrote and edited the manuscript. All authors read and approved the final manuscript.

## Supplementary Material

Additional file 1 **Workflow**. Demonstration of the entirely process, including fracture reduction and fixation. Click here for file

## References

[B1] Rommens PM Acetabulum fractures Unfallchirurg 1999 102 589 590 10.1007/s001130050454 10484902

[B2] Hirvensalo E Lindahl J Kiljunen V Modified and new approaches for pelvic and acetabular surgery Injury 2007 38 431 441 10.1016/j.injury.2007.01.020 17445529

[B3] Letournel E The treatment of acetabular fractures through the ilioinguinal approach Clin Orthop Relat Res 1993 62 76 8519138

[B4] Jimenez ML Vrahas MS Surgical approaches to the acetabulum Orthop Clin North Am 1997 28 419 434 10.1016/S0030-5898(05)70299-6 9208834

[B5] Matta JM Operative treatment of acetabular fractures through the ilioinguinal approach: a 10-year perspective J Orthop Trauma 2006 20 S20 29 16385203

[B6] Hirvensalo E Lindahl J Bostman O A new approach to the internal fixation of unstable pelvic fractures Clin Orthop Relat Res 1993 28 32 8242945

[B7] Cole JD Bolhofner BR Acetabular fracture fixation via a modified Stoppa limited intrapelvic approach. Description of operative technique and preliminary treatment results Clin Orthop Relat Res 1994 112 123 8050220

[B8] Ponsen KJ Joosse P Schigt A Goslings JC Luitse JS Internal fracture fixation using the Stoppa approach in pelvic ring and acetabular fractures: technical aspects and operative results J Trauma 2006 61 662 667 10.1097/01.ta.0000219693.95873.24 16967004

[B9] Crowl AC Kahler DM Closed reduction and percutaneous fixation of anterior column acetabular fractures Comput Aided Surg 2002 7 169 178 10.3109/10929080209146027 12362377

[B10] Gross T Jacob AL Messmer P Regazzoni P Steinbrich W Huegli RW Transverse acetabular fracture: hybrid minimal access and percutaneous CT-navigated fixation AJR Am J Roentgenol 2004 183 1000 1002 15385293 10.2214/ajr.183.4.1831000

[B11] Giannoudis PV Tzioupis CC Pape HC Roberts CS Percutaneous fixation of the pelvic ring: an update J Bone Joint Surg Br 2007 89 145 154 10.1302/0301-620X.89B2.18551 17322425

[B12] Rommens PM Is there a role for percutaneous pelvic and acetabular reconstruction? Injury 2007 38 463 477 10.1016/j.injury.2007.01.025 17397846

[B13] Ohashi K El-Khoury GY Abu-Zahra KW Berbaum KS Interobserver agreement for Letournel acetabular fracture classification with multidetector CT: are standard Judet radiographs necessary? Radiology 2006 241 386 391 10.1148/radiol.2412050960 17005769

[B14] Geijer M El-Khoury GY Imaging of the acetabulum in the era of multidetector computed tomography Emerg Radiol 2007 14 271 287 10.1007/s10140-007-0638-5 17588182

[B15] Scott WW Jr Magid D Fishman EK Riley LH Jr Brooker AF Jr Johnson CA Three-dimensional imaging of acetabular trauma J Orthop Trauma 1987 1 227 232 10.1097/00005131-198701030-00006 3506056

[B16] Brown GA Milner B Firoozbakhsh K Application of computer-generated stereolithography and interpositioning template in acetabular fractures: a report of eight cases J Orthop Trauma 2002 16 347 352 10.1097/00005131-200205000-00010 11972079

[B17] Citak M Gardner MJ Kendoff D Tarte S Krettek C Nolte LP Hufner T Virtual 3D planning of acetabular fracture reduction J Orthop Res 2008 26 547 552 10.1002/jor.20517 17972324

[B18] Cimerman M Kristan A Preoperative planning in pelvic and acetabular surgery: the value of advanced computerised planning modules Injury 2007 38 442 449 10.1016/j.injury.2007.01.033 17400226

[B19] Hege HC Seebass M Stalling D A Generalized Marching Cubes Algorithm Based on Non-Binary Classifications Konrad-Zuse-Zentrum für Informationstechnik Berlin; Technical Report 1997

[B20] Fornaro J Harders M Keel M Marincek B Trentz O Szekely G Frauenfelder T Interactive visuo-haptic surgical planning tool for pelvic and acetabular fractures Stud Health Technol Inform 2008 132 123 125 18391271

[B21] Letournel E Acetabulum fractures: classification and management Clin Orthop Relat Res 1980 81 106 7418327

[B22] Dakin GJ Eberhardt AW Alonso JE Stannard JP Mann KA Acetabular fracture patterns: associations with motor vehicle crash information J Trauma 1999 47 1063 1071 10.1097/00005373-199912000-00012 10608534

[B23] Vanderschot P Treatment options of pelvic and acetabular fractures in patients with osteoporotic bone Injury 2007 38 497 508 10.1016/j.injury.2007.01.021 17399713

